# Supplementary material for: Limosilactobacillus fermentum Strain 3872: Antibacterial and Immunoregulatory Properties and Synergy with Prebiotics against Socially Significant Antibiotic-Resistant Infections of Animals and Humans
Source: Antibiotics (Basel). 2022 Oct 19;11(10):1437. doi: 10.3390/antibiotics11101437 (PMC9598557; doi:10.3390/antibiotics11101437)
Supplement: Supplementary file 1 [file antibiotics-11-01437-s001.zip › antibiotics-1965829-supplementary.pdf]

Supplementary Materials

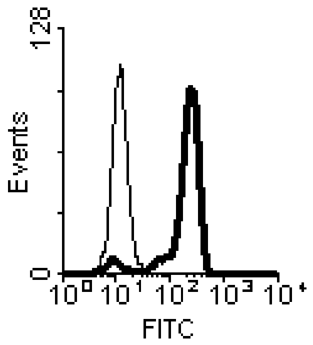

Figure S1. CD14 expression on the surface of primary human peripheral blood monocytes according to flow cytometry. Note: FITC-labeled cells are shown on the histogram with a bold line; isotypic control is shown on the histogram with a thin line.

```
CLUSTAL O(1.2.4) multiple sequence alignment


zoocin_A                           GHVGVDYA--VPVGTPVRAVANGTVKFAGNGANHPWMLWMAGNCV--LIQHADGMHTGYA56
zoocin_A_peptidase_family_M23      FHAGFDLKTNQREGLNVYAVADGYVSRIK------ISTFGNGKCI--YVTHPNGYTSVYG52
BLF3872                            -HDGWDFQQATWGGQNVVAVHDGTVYKVA------YSSDGRDWHV--DVKSDDGWYETYQ51
enterolysin_A                      FHDGFDFGSAIYGNGSVYAVHDGKILYAG------WDPVGGGSLGAFIVLQAGNTNVIYQ54
                                    * * *       .  * ** :* :                .      :   ..    *

zoocin_A                           HL----SKISVSTDSTVKQGQIIGYTGATGQVTGPHLHFEMLP-----------------95
zoocin_A_peptidase_family_M23      HLQT--------------------------------------------------------56
BLF3872                            EGFLSKSDIAVKVGDKVKVGDKI------GTLTGTHLHLGVSKTEIEKAQSSWNKDDGTW105
enterolysin_A                      EFSRNVGDIKVSTGQTVKKGQLI------GKFTSSHLHLGMTKKE---------------93
                                   .

zoocin_A                           ------------------- 95
zoocin_A_peptidase_family_M23      ------------------- 56
BLF3872                            KNPLDIISGGGSSDSSSPK 124
enterolysin_A                      ------------------- 93
```

Figure S2. Alignment of sequences of class III bacteriocins: BLF3872, enterolysin A from *E. faecalis* LMG 2333, zoocin A from *S. zooepidemicus*, and zoocin A peptidase family M23 from *F. johnsoniae* (Clustal Omega program (https://www.ebi.ac.uk/Tools/msa/clustalo/), accessed on 27 September 2022). Identical, conserved and semi-conserved residues are denoted as "*", ":" and "." respectively.

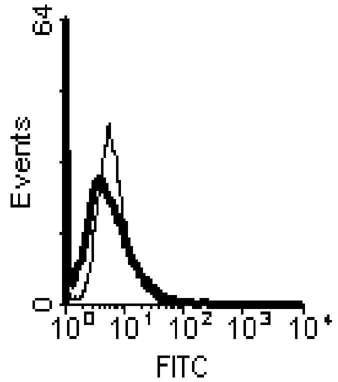

Figure S3. Absence of CD14 expression on the surface of IDC derived from human peripheral blood monocytes. Note: FITC-labeled cells are shown on the histogram with a bold line; isotypic control is shown on the histogram with a thin line.
